# Comparison of the Effect of Three Cements on Prevention of Enamel Demineralization Adjacent to Orthodontic Bands

**DOI:** 10.5681/joddd.2012.019

**Published:** 2012-09-01

**Authors:** Mehdi Kashani, Sareh Farhadi, Neda Rastegarfard

**Affiliations:** ^1^Assistant Professor, Department of Orthodontics, Faculty of Dentistry, Shahed University, Tehran, Iran; ^2^Assistant Professor, Department of Oral and Maxillofacial Pathology, Faculty of Dentistry, Shahed University, Tehran, Iran; ^3^Undergraduate Student, Faculty of Dentistry, Shahed University, Tehran, Iran

**Keywords:** Cement, demineralization, dye penetration, orthodontic band

## Abstract

**Background and aims:**

This in vitro study was designed to compare enamel demineralization depths adjacent to bands cemented with zinc polycarboxylate, glass ionomer (GI) and resin-modified glass ionomer (RMGI), in order to achieve minimal enamel demineralization during orthodontic treatment.

**Materials and methods:**

Sixty fully developed extracted third molars were randomly divided into three testgroups each containing 20 samples, used to cement orthodontic bands with zinc polycarboxylate, GI and RMGI. All samples were demineralized using White method using hydroxyapatite, latic acid and Carbapol for in vitro caries simulation, and then, immersed in 10% solution of methylene blue. The mean depth of dye penetration was assessed up to 0.1 millimeter, reflect-ing the depth of enamel demineralization. One way ANOVA and LSD statistical tests were employed to evaluate significant differences among groups.

**Results:**

The highest dye penetration depth was seen in zinc polycarboxylate group, followed by GI, and RMGI groups, respectively, with significant differences among each two groups (P < 0.05).

**Conclusion:**

The use of RMGI cement seems to present significantly better prevention of enamel demineralization adja-cent to orthodontics bands.

## Introduction

Enamel demineralization adjacent to bands and brackets is a great complication in the patients of fixed orthodontic treatment especially those with poor oral hygiene.^1 - 3^ Demineralization takes place when specific bacteria are retained for a long time on the enamel surface. The bacteria metabolize carbohydrates and produce organic acids, dissolving the calcium phosphate mineral of tooth structures.^4^ Different studies have reported the prevalence of these lesions up to 95% during fixed orthodontic treatment.^5 - 7^ Demineralization can occur within few weeks,^8^ a length of time that is usually shorter than preferred by most orthodontists.^9 , 10^ It has been generally accepted that, the combined application of fluoride regimens, oral hygiene instructions and dietary control can contribute greatly to inhibition of demineralization during fixed appliance treatment. However, these preventive strategies need patient compliance,^11 - 16^ and hence, are not reliable.^17 - 19^

Therefore, preventive strategies which do not need the patient’s compliance might be more effective in preventing or reducing demineralization.^20 , 21^ The professional application of fluoride varnish has been shown to be effective in this situation.^22^ Fluoride varnish adheres to enamel surface longer than other topical fluoride products and has been shown to be superior because of its ability to increase fluoride uptake in enamel in vitro.^23^ Also, fluoride has been added to the materials used for cementation of bands and brackets. The cement acts as an additional local source of fluoride near the appliances, whereas the amount of release depends on the substance group each material belongs to. ^1, 24^

Conventional glass ionomer cement has been evaluated for bonding orthodontic brackets due to its anti-cariogenic property attributed to release of fluoride.^2^ Resin- modified glass ionomer cements have higher adhesive strength than conventional glass ionomer and can also control caries around orthodontic appliances. ^26 , 27^ Although previous studies have evaluated the efficacy of different cements, few have compared the efficacy of different routinely-used materials for cementation of orthodontic appliances. ^28^ Therefore, the present study was designed to compare zinc polycarboxylate, conventional glass ionomer, and resin-modified glass ionomer cements with regard to enamel demineralization adjacent to orthodontic bands after in vitro pH cycling.

## Materials and Methods

Fully developed extracted third molars with same morphology and size were included in this study. The teeth were collected from Department of Oral & Maxillofacial Surgery, Faculty of Dentistry, Shahed University, and two private dental clinics in Tehran, Iran. Written consents were obtained from all patients. The teeth were assessed precisely and those with enamel hypoplasia, developmental malformations, discolorations and any clinical evidence of dental caries were excluded from study. Then, the sixty selected teeth were cleaned with fluoride-free pumice by low-speed handpiece to remove any debris, rinsed with deionized water, and randomly divided into three test groups, each containing 20 samples. All teeth were embedded in acrylic blocks.

Stainless steel microethced orthodontic bands (IMD, China) with attachments were fitted on teeth, and margins were adapted by band pusher. The bands were approximately seated at the same position of each tooth on middle third part of crowns. Then, bands were tightly fitted to decrease the possibility of enamel dissolution. After manipulating, the bands were cemented on teeth in each group using one the following materials according to the manufacturers’ instructions: zinc polycarboxylate cement (Harvard Dental International, Gmbh, Germany); glass ionomer (GI) cement (Fuji GC, Japan); resin-modified glass ionomer (RMGI) cement (Biodinamica, Brazil). RMGI was polymerized with curing light, from two opposite sides of the bands for 40 seconds, to ensure adequate polymerization. In all samples, additional cement was removed before polymerization from occlusal and cervical margins of the bands to prevent affecting test results. The cements were allowed to bench set for 2 minutes at a uniform ambient temperature of 25ºC. Then, to prevent demineralization of tooth body, each tooth was coated with two layers of nail varnish, except a 2×2 mm window under cervical margin of the band, which was designated for evaluation of demineralization.^18^ In this manner, the enamel both directly in contact with cement and up to 2 mm away from the cement had similar situation for induction of in vitro demineralization. Therefore, it would be an internal control for testing of in vitro pH cycling performance.

**Figure 1:**
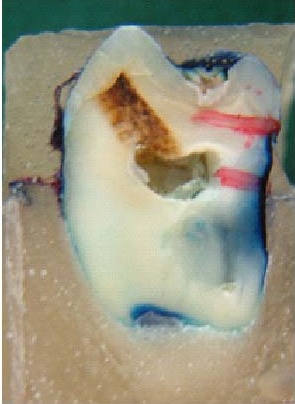
Sample sectioned buccolingually through the midline of exposed window by disk.

In vitro caries was created using the method of White,^12^ as follows: the samples were demineralized in 10 mL of an acidic solution (pH = 4.8) at 37°C, containing 500 mg per liter of hydroxyapatite, 0.1 mol per liter of lactic acid and 20 g per liter of carbopol 907; the solution was changed every 4 hours for 96 hours.^12^ Then, they were removed and rinsed with deionized water. The use of colored agents to demonstrate microleakage continues to be the most popular of techniques currently available. This method allows the production of sections showing leakage in contrasting colors to the tooth and without the need for further chemical reaction or exposure to potentially hazardous radiation.^29^ So, in the next step, teeth were immersed in a 10% solution of methylene blue at 37°C to evaluate the created demineralization through dye penetration.^11^ By then, the teeth were removed and rinsed with deionized water. After removing bands by band remover, samples were sectioned buccolingually through the midline of exposed window by disk (Isomet low speed saw and wafering blades) (Figure 1).

**Figure 2:**
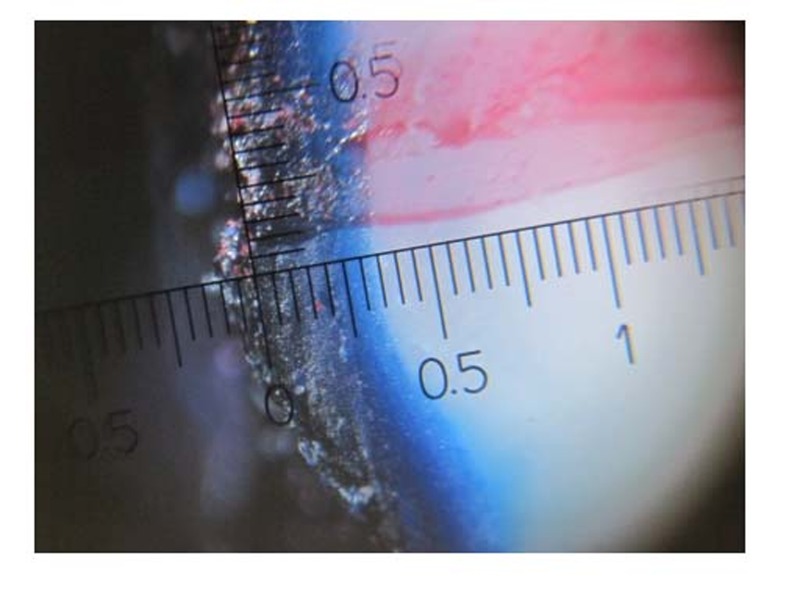
Stereomicroscopic view of methylene-blue penetration (×80 magnification). The area between arrows points to enamel demineralization region.

Imbibition of a dye into porosities of demineralized enamel enables quantitative analysis of demineralization by stereomicroscope (Ruler-JY 80X) with ×80 magnification (Figure 2). The depths of dye penetration in specimens were evaluated by two examiners up to 0.1 millimeter, and the average of examiners’ measurements for each sample was recorded.

### Statistical Analysis

Descriptive statistics, including means and standard deviations, were calculated for dye penetration in all study groups. Then, results were analyzed by one-way ANOVA to detect significant differences among all groups. Then, multiple comparison LSD tests were applied to identify significant differences for dye penetration between each two groups. The statistical significance level was set at P < 0.05.

## Results

The mean dye penetration of all groups is given in Table 1. The highest demineralization depth was seen in polycarboxylate group, followed by GI and RMGI groups, respectively. One way ANOVA test showed significant differences between all groups (P < 0.000). The results of LSD tests was also statistically significant (P < 0.000) between each two groups (Table 2).

**Table 1:** The mean dye penetration in the study groups (n = 20)

Cement	N	Mean ± SD (mm)
Polycarboxylate	20	1.51±0.35
glass ionomer	20	0.81±0.23
RMGI	20	0.41±0.15

**Table 2:** The results of two by two comparisons of study groups

Cement	Zinc polycarboxylate	GI	RMGI
Zinc polycarboxylate	—	—	—
GI	0.70*	—	—
RMGI	1.10*	0.40*	—

* Statistically significant (P <0.05)

## Discussion

Clinical experience shows that the orthodontic band makes tooth more prone to decalcification. This is because the orthodontic band with its welded attachment provides a place for plaque accumulation and when the band is not fully fitted onto the tooth, there will be a gap between the band and the tooth which makes the tooth more prone to decalcification. Studies have shown 85% of the cervical and occlusal margins of the band present cement defects.^30^ Therefore, using fluoride releasing cements for band cementation would be effective in preventing enamel decalcification.

In this study, there were significant differences among demineralization depths of zinc polycarboxylate, GI and RMGI groups. The differences might be justified by the higher amount of fluoride released by RMGI as well as its appropriate bond strength and lower solubility. ^11 , 21 , 31^ The lowest demineralization observed around orthodontic bands cemented with RMGI is consistent with the results previous studies.,^3^ Therefore, considering the use of RMGI as a preventive orthodontic banding cement might be recommended pending further investigations.

The highest demineralization adjacent to bands was seen in zinc polycarboxylate group (P<0.05). Evaluation of demineralization adjacent to zinc polycarboxylate cement has been done before,^11 , 34^ with findings similar to those of the present study. High demineralization around this cement might be related to its physical properties, as research on zinc polycarboxylate cement has shown poor bond strength to enamel and dentin as well as high solubility.^3^ Although the experimental protocol was similar to those in previous studies, an attempt was made to provide a similar situation for all groups in the present study to reduce the effect of these factors. Indeed, the time taken for causing demineralization was as long as 3 months of real time,^12^ compare to most studies. However, with regards to studies showing the high amount of fluoride releasing in first three days from cementing and then a substantial decrease after the third week,^19^ evaluating the effect of these cements for longer period would not be efficient. Therefore, we tried to improve the reliability of the study by increasing the volume of the samples, compared with previous studies.

Although in vitro studies can be helpful in evaluating the demineralization properties of polycarboxylate, glass ionomer and resin-modified glass ionomer cements, laboratory conditions cannot replicate the exact clinical condition. Further controlled clinical trial is warranted.

## Conclusion

With regard to limitation of an in vitro setting, it can be concluded that the use of RMGI would provide significantly better prevention of enamel demineralization adjacent to orthodontic bands.
